# Temporal trends in bystander cardiopulmonary resuscitation and favourable neurological outcome among older patients with out-of-hospital cardiac arrest stratified by bystander–patient relationship and age: a JCS-ReSS study

**DOI:** 10.1016/j.resplu.2026.101525

**Published:** 2026-10-07

**Authors:** Akira Funada, Yoshikazu Goto, Masayuki Takamura, Naohiro Yonemoto, Tetsuya Matoba, Yoshio Tahara

**Affiliations:** a Department of Cardiology, Osaka Saiseikai Senri Hospital, 1-1-6 Tsukumodai, Suita, Osaka 565-0862, Japan; b Department of Emergency and Critical Care Center, Noto General Hospital, 6-4 A-bu, Fujihashi-machi, Nanao, Ishikawa 926-0816, Japan; c Department of Cardiology, Kanazawa University Graduate School of Medicine, Kanazawa University, 13-1 Takara-machi, Kanazawa, Ishikawa 920-8641, Japan; d Department of Biostatistics, School of Medicine, University of Toyama, 2630 Sugitani, Toyama, Toyama 930-0194, Japan; e Department of Cardiovascular Medicine, Kyushu University, 3-1-1 Maidashi, Higasi-Ku, Fukuoka, Fukuoka 812-8582, Japan; f Department of Cardiovascular Medicine, National Cerebral and Cardiovascular Center, 6-1 Kishibe-shinmachi, Suita, Osaka 564-8565, Japan

**Keywords:** Out-of-hospital cardiac arrest, Elderly, Bystander cardiopulmonary resuscitation, Neurological outcome, Temporal trend

## Abstract

**Aim:**

To describe temporal trends in bystander cardiopulmonary resuscitation (CPR) and favourable neurological outcomes among older patients with bystander-witnessed out-of-hospital cardiac arrest (OHCA) according to bystander–patient relationship and age.

**Methods:**

A total of 322,943 patients aged ≥65 years with bystander-witnessed OHCA of cardiac origin were enrolled from the All-Japan Utstein Registry (2005–2022). Patients were stratified by age (65–74, 75–89, and ≥90 years) and bystander type (family member, friend/colleague/passerby, or other). Temporal trends in the proportions of bystander CPR and favourable neurological outcome at 1-month, defined as Cerebral Performance Category (CPC) 1–2, were assessed using the Cochran–Armitage test.

**Results:**

During the study period, the annual proportions of bystander CPR and CPC 1–2 in family member-witnessed OHCAs significantly increased across all age groups (all *p* for trend <0.001). However, bystander CPR remained at approximately 50% in all age groups. In other-witnessed OHCAs, bystander CPR also significantly increased across all age groups (all *p* for trend <0.001). However, CPC 1–2 significantly increased only in those aged 65–74 years (*p* for trend <0.05), with no significant temporal trend in older groups. Among those aged ≥90 years, bystander CPR increased to approximately 80%, whereas CPC 1–2 remained at approximately 1%, with no significant temporal trend.

**Conclusions:**

Temporal trends in bystander CPR and CPC 1–2 varied according to bystander–patient relationship and age. Further investigation is warranted to identify the factors underlying the observed differences in bystander CPR and favourable neurological outcomes.

## Highlights

• Bystander CPR and CPC 1–2 increased over time in family-witnessed OHCA in Japan.
• However, bystander CPR remained at ∼50% in family-witnessed OHCA.
• Bystander CPR reached ∼80% in other-witnessed OHCA aged ≥90 years.
• However, CPC 1–2 was about 1% in other-witnessed OHCA aged ≥90 years.
• Temporal trends varied by bystander–patient relationship and age.

## Introduction

Japan is one of the countries with a super-aged population. With population aging, the number of emergency calls involving older patients has increased, leading to more hospital transports for older patients with out-of-hospital cardiac arrest (OHCA).^1^, ^2^, ^3^ In Japan, emergency medical services (EMS) personnel are legally prohibited from terminating resuscitation efforts in the field; therefore, most older patients with OHCA who receive cardiopulmonary resuscitation (CPR) from EMS personnel are transported to hospitals.^4^ However, medical resources are limited, and increasing demands for emergency care may place an additional burden on the healthcare system. Although the introduction of termination of resuscitation criteria in the emergency setting may be desirable, ethical considerations currently hinder their implementation in Japan.^5^, ^6^ In addition, advance care planning (ACP), including discussions about preferences regarding resuscitation, has not progressed sufficiently among older patients in primary care settings.^7^, ^8^ EMS workload and in-hospital treatment appear to be approaching their operational limits within the chain of survival.^9^

Nevertheless, there remains room for improvement in the provision of bystander CPR in the prehospital setting.^10^, ^11^, ^12^, ^13^ Although neurological outcomes among older patients with OHCA are generally poor, bystander CPR is provided uniformly in Japan irrespective of age; moreover, the proportion of bystander CPR tends to be higher among older patients.^1^, ^3^, ^4^, ^14^ Previous studies have reported differences in the receipt of bystander CPR and neurological outcomes according to the bystander–patient relationship.^15^, ^16^, ^17^, ^18^ Understanding temporal changes in the receipt of bystander CPR and favourable neurological outcomes may help improve prehospital resuscitation and inform future discussions about resuscitation preferences in an aging population.

This study aimed to describe temporal trends in the receipt of bystander CPR and favourable neurological outcomes among older patients with OHCA according to bystander–patient relationship and age.

## Methods

### Study design and data source

In January 2005, the Fire and Disaster Management Agency (FDMA) of Japan launched a prospective, nationwide, population-based registry based on Utstein-style data collection that included all patients with OHCA (the All-Japan Utstein registry).^19^, ^20^, ^21^, ^22^, ^23^ This observational study included patients aged ≥65 years with OHCA of presumed cardiac origin, witnessed by a citizen bystander, between January 2005 and December 2022. The data analysed in this study were curated and provided through the Japanese Circulation Society–Resuscitation Science Study (JCS-ReSS) Group. Cardiac arrest is defined by the absence of signs of circulation.^19^ Cause of cardiac arrest was determined by the physicians in charge and EMS personnel.

This study was approved by the Ethics Committee of Osaka Saiseikai Senri Hospital (Approval No. 070404), and the requirement for informed consent was waived due to the anonymous nature of the data.

### Japanese EMS system

Japan has approximately 125 million residents living in an area of 378,000 km^2^. Details of the Japanese EMS system have been described previously.^20^, ^21^, ^22^, ^23^ The FDMA of Japan supervises the nationwide EMS system, while local fire stations operate the local EMS services. Emergency lifesaving technicians (ELSTs) are authorized to use automated external defibrillators (AEDs), insert an airway adjunct or a peripheral intravenous line, and administer Ringer’s lactate solution. Only specially trained ELSTs are permitted to insert a tracheal tube and administer intravenous adrenaline after receiving instructions from physician via telephone. All EMS personnel perform CPR according to the Japanese CPR guidelines. The latest guidelines were updated in 2020.^4^ As EMS providers in Japan are legally prohibited from terminating resuscitation efforts in the field, most OHCA patients who receive CPR by EMS personnel are transported to hospital, except in cases of certain fatality.

### Data collection

Data were collected prospectively for variables including age, gender, cause of arrest, bystander-witness status, bystander CPR with or without AED, initial cardiac rhythm and bystander type. Other variables included whether adrenaline was administered, advanced airway management techniques were used and return of spontaneous circulation (ROSC) was achieved before hospital arrival. Timing variables were also recorded, including the times of witnessed collapse, emergency call, ambulance arrival at the scene, CPR initiation by EMS personnel, ROSC, ambulance arrival at the hospital, adrenaline administration, and shock delivery by EMS personnel. Moreover, information on survival and neurological outcomes at 1-month after cardiac arrest was collected. Neurological outcomes were defined using the Cerebral Performance Category (CPC) scale: category 1, good cerebral performance; 2, moderate cerebral disability; 3, severe cerebral disability; 4, coma or vegetative state; and 5, death.^19^ Categorization was performed by the physician in charge.

### Outcome

The primary outcomes were receipt of bystander CPR and favourable neurological outcome at 1-month, defined as a CPC score of 1 or 2 (CPC 1–2).

### Statistical analysis

Continuous variables are presented as means ± standard deviations and medians (interquartile ranges) and compared using the Kruskal–Wallis tests. Categorical variables are presented as counts and percentages, and differences between groups were assessed using the *χ*^2^ test. Patients were categorized into three age groups (65–74, 75–89, or ≥90 years) according to the latest classification of older adults in Japan.^24^ Bystanders were categorized as family member, friend, colleague, passerby, or other according to the All-Japan Utstein Registry. For analysis, friend, colleague, and passerby were combined into a single group, resulting in three bystander type groups for comparison: family member, combined friend/colleague/passerby, and other.^17^ Annual trends in the proportions of patients receiving bystander CPR and achieving CPC 1–2 at 1-month were analysed using the Cochran–Armitage test for trends, stratified by bystander type and age group. All statistical analyses were performed using JMP Student Edition, version 18.2.2 (SAS Institute, Cary, NC, USA). All tests were two-tailed, and a *p* value < 0.05 was considered statistically significant.

## Results

During the 18-year study period, a total of 2,202,145 OHCA events were documented. After excluding patients with no resuscitation attempt by EMS personnel (*n* = 48,434), non-cardiac origin (*n* = 875,341), unwitnessed arrest (*n* = 763,230), EMS-witnessed arrest (*n* = 99,546), unknown witness status (*n* = 3643), age <65 years or unknown (*n* = 84,954), unknown bystander CPR status (*n* = 4046), and unknown 1-month outcome (*n* = 8), 322,943 older patients (aged ≥65 years) with OHCA of presumed cardiac origin witnessed by a citizen bystander were included in this study.

The baseline characteristics and outcomes by age group are shown in Table 1. With increasing age, the proportion of male patients decreased, the proportion of bystander CPR increased, the proportion of shockable rhythms declined, EMS interventions such as defibrillation, administration of adrenaline, and advanced airway management decreased, and neurological outcomes were poorer. The proportions of patients according to bystander type by age group are presented in Fig. 1. The proportion of family member was highest among patients aged 75–89 years, that of combined friend/colleague/passerby among those aged 65–74 years, and that of other among those aged ≥90 years. Family member had the highest proportion across all age groups. The proportions of family member and the combined friend/colleague/passerby decreased with increasing age, whereas the proportion of other increased (all *p* for trend <0.001). The number of patients aged 65–74 years remained stable, whereas the number of those aged 75–89 years increased 1.81-fold (from 7144 in 2005 to 12,912 in 2022), and those aged ≥90 years increased 2.91-fold (from 1927 in 2005 to 5608 in 2022) (Fig. 2A).

**Table 1:** Baseline patient characteristics and post-cardiac arrest outcomes in all patients and by age group.

**Variable**	**All patients**	**Age group**			***P* value**
		**65–74 years**	**75–89 years**	**≥90 years**	
	***n* = 322,943**	***n* = 78,022**	***n* = 182,158**	***n* = 62,763**	
**Age (years)**					
Mean ± SD	81.5 ± 8.6	69.9 ± 2.8	82.3 ± 4.2	93.5 ± 3.1	
Median, IQR	82 (75–88)	70 (68–72)	82 (79–86)	93 (91–95)	<0.001
Male	183,869 (56.9)	56,943 (73.0)	106,235 (58.3)	20,691 (33.0)	<0.001
**Bystander type (witnessed by)**					
Family member	211,398 (65.5)	52,647 (67.5)	124,558 (68.4)	34,193 (54.5)	<0.001
Combined friend/colleague/passerby	24,448 (7.6)	12,657 (16.2)	10,711 (5.9)	1080 (1.7)	
Other	87,097 (27.0)	12,718 (16.3)	46,889 (25.7)	27,490 (43.8)	
Bystander CPR	167,922 (52.0)	37,655 (48.3)	92,888 (51.0)	37,379 (59.6)	<0.001
Public-access defibrillation performed by bystander	5895 (1.8)	2326 (3.0)	2672 (1.5)	897 (1.4)	<0.001
**Initial documented cardiac arrest rhythm**					
Shockable rhythm (VF or pulseless VT)	45,595 (14.1)	21,306 (27.3)	21,373 (11.7)	2916 (4.6)	<0.001
Non-shockable rhythm (Asystole or PEA)	271,094 (83.9)	55,045 (70.6)	157,345 (86.4)	58,704 (93.5)	
Unidentified	6254 (1.9)	1671 (2.1)	3440 (1.9)	1143 (1.8)	
Shock delivery by EMS personnel	60,146 (18.6)	26,134 (33.5)	29,333 (16.1)	4679 (7.5)	<0.001
Adrenaline administration	84,430 (26.1)	21,851 (28.0)	48,960 (26.9)	13,619 (21.7)	<0.001
Advanced airway management use	152,805 (47.3)	39,235 (50.3)	88,122 (48.4)	25,448 (40.5)	<0.001
**Time interval from collapse to emergency call (min) (*n* = 315,185)**					
Mean ± SD	3.2 ± 7.5	2.7 ± 6.8	3.3 ± 7.6	3.3 ± 7.8	
Median, IQR	2 (0–5)	2 (0–4)	2 (0–5)	2 (0–5)	<0.001
**Time interval from collapse to initiation of bystander CPR (min) (*n* = 164,281)**					
Mean ± SD	3.7 ± 5.9	3.7 ± 5.4	3.9 ± 6.1	3.3 ± 5.7	
Median, IQR	2 (0–5)	2 (0–5)	2 (0–5)	1 (0–5)	<0.001
**Time interval from collapse to first contact with EMS provider (min) (*n* = 318,649)**					
Mean ± SD	12.1 ± 7.8	11.6 ± 7.3	12.2 ± 7.9	12.3 ± 8.0	
Median, IQR	11 (7–15)	10 (7–14)	11 (7–15)	11 (7–15)	<0.001
Prehospital ROSC	40,859 (12.7)	13,933 (17.9)	22,083 (12.1)	4843 (7.7)	<0.001
**Outcomes**					
Survival at 1-month	22,523 (7.0)	10,607 (13.6)	10,598 (5.8)	1318 (2.1)	<0.001
CPC 1–2 at 1-month	113,65 (3.5)	6197 (7.9)	4689 (2.6)	479 (0.8)	<0.001

Values are number of patients (%) unless indicated otherwise. CPC indicates Cerebral Performance Category scale; CPR, Cardiopulmonary resuscitation; EMS, Emergency medical services; IQR, Interquartile range; OHCA, Out-of-hospital cardiac arrest; PEA, Pulseless electrical activity; ROSC, Return of spontaneous circulation; SD, Standard deviation; VF, Ventricular fibrillation; VT, Ventricular Tachycardia.

**Fig. 1:**
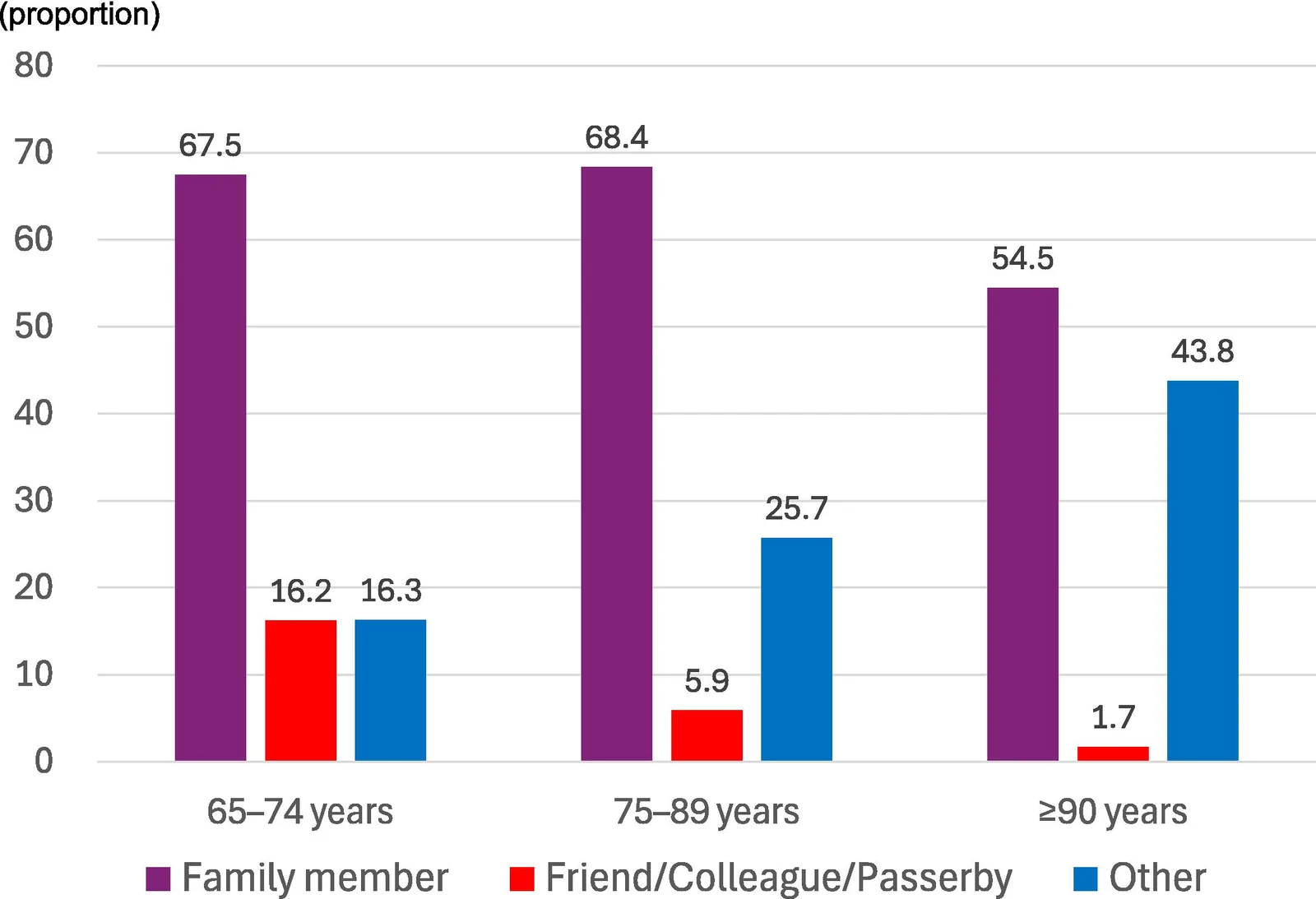
**Proportion of patients with OHCA by bystander type across age groups**.

**Fig. 2:**
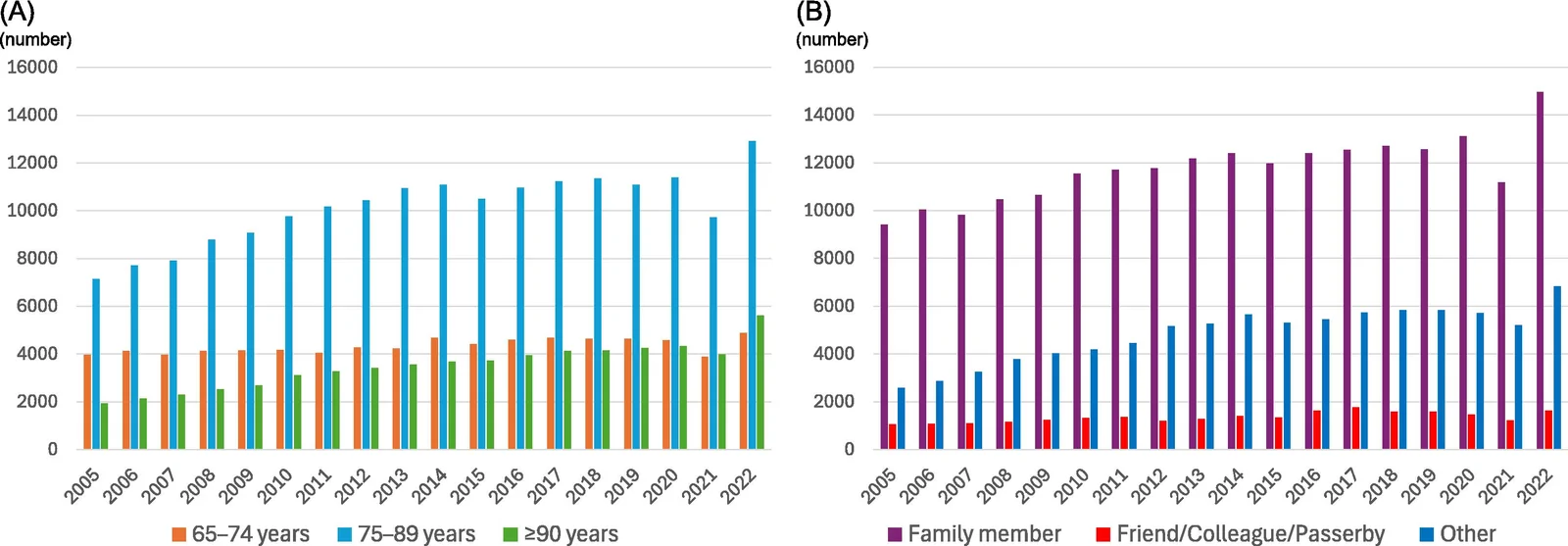
**Annual trends in the number of patients with OHCA by (A) age group and (B) bystander type**.

The baseline characteristics and outcomes according to bystander type are presented in Table 2. The combined friend/colleague/passerby group was younger, whereas other group was older. Other group had a lower proportion of male patients and a higher proportion of patients receiving bystander CPR. Public-access defibrillation was less frequent in family member group, whereas shockable rhythms were less frequent in other group. EMS interventions were less common in other group. Calls made prior to collapse were more frequent, and the time from collapse to bystander CPR was shorter in other group. Compared with the combined friend/colleague/passerby group, neurological outcomes were poorer among family member or other groups. The number of patients witnessed by the combined friend/colleague/passerby group remained stable, whereas the number of those witnessed by family member and other groups increased (Fig. 2B).

**Table 2:** Baseline patient characteristics and post-cardiac arrest outcomes in all patients and by bystander type.

**Variable**	**All patients**	**Bystander type (Witnessed by)**			***P* value**
		**Family member**	**Combined** **friend/colleague/passerby**	**Other**	
	***n* = 322,943**	***n* = 211,398**	***n* = 24,448**	***n* = 87,097**	
**Age (years)**					
Mean ± SD	81.5 ± 8.6	80.9 ± 8.3	75.4 ± 7.5	84.6 ± 8.5	
Median, IQR	82 (75–88)	81 (75–87)	74 (69–80)	86 (79–91)	<0.001
**Age group**					
65–74 years	78,022 (24.2)	52,647 (24.9)	12,657 (51.8)	12,718 (14.6)	<0.001
75–89 years	182,158 (56.4)	124,558 (58.9)	10,711 (43.8)	46,889 (53.8)	
≥90 years	62,763 (19.4)	34,193 (16.2)	1080 (4.4)	27,490 (31.6)	
Male	183,869 (56.9)	128,890 (61.0)	17,831 (72.9)	37,148 (42.7)	<0.001
Bystander CPR	167,922 (52.0)	90,527 (42.8)	12,346 (50.5)	65,049 (74.7)	<0.001
Public-access defibrillation performed by bystander	5895 (1.8)	586 (0.3)	1564 (6.4)	3745 (4.3)	<0.001
**Initial documented rhythm**					
Shockable rhythm (VF or pulseless VT)	45,595 (14.1)	29,187 (13.8)	8146 (33.3)	8262 (9.5)	<0.001
Non-shockable rhythm (Asystole or PEA)	271,094 (83.9)	179,282 (84.8)	15,606 (63.8)	76,206 (87.5)	
Unidentified	6254 (1.9)	2929 (1.4)	696 (2.8)	2629 (3.0)	
Shock delivery by EMS personnel	60,146 (18.6)	39,153 (18.5)	9683 (39.6)	11,310 (13.0)	<0.001
Adrenaline administration	84,430 (26.1)	57,775 (27.3)	7015 (28.7)	19,640 (22.6)	<0.001
Advanced airway management use	152,805 (47.3)	105,189 (49.8)	11,632 (47.6)	35,984 (41.3)	<0.001
**Time interval from collapse to emergency call (min) (*n* = 315,185)**					
Mean ± SD	3.2 ± 7.5	3.5 ± 7.7	2.1 ± 5.8	2.8 ± 7.3	
Median, IQR	2 (0–5)	2 (0–5)	1 (0–4)	2 (−1–5)	<0.001
**Time interval from collapse to initiation of bystander CPR (min) (*n* = 164,281)**					
Mean ± SD	3.7 ± 5.9	5.0 ± 6.6	3.1 ± 4.5	2.1 ± 4.4	
Median, IQR	2 (0–5)	3 (1–6)	2 (0–5)	0 (0–2)	<0.001
**Time interval from collapse to first contact with EMS provider (min) (*n* = 318,649)**					
Mean ± SD	12.1 ± 7.8	12.4 ± 8.0	10.8 ± 6.9	11.6 ± 7.6	
Median, IQR	11 (7–15)	11 (8–15)	10 (7–13)	10 (7–15)	<0.001
Prehospital ROSC	40,859 (12.7)	25,203 (11.9)	5199 (21.3)	10,457 (12.0)	<0.001
**Outcomes**					
Survival at 1-month	22,523 (7.0)	13,089 (6.2)	4389 (18.0)	5045 (5.8)	<0.001
CPC 1–2 at 1-month	113,65 (3.5)	6016 (2.8)	2723 (11.1)	2626 (3.0)	<0.001

Values are number of patients (%) unless indicated otherwise. CPC indicates Cerebral Performance Category scale; CPR, Cardiopulmonary resuscitation; EMS, Emergency medical services; IQR, Interquartile range; OHCA, Out-of-hospital cardiac arrest; PEA, Pulseless electrical activity; ROSC, Return of spontaneous circulation; SD, Standard deviation; VF, Ventricular fibrillation; VT, Ventricular Tachycardia.

Fig. 3A–C presents the annual trends in the proportion of patients receiving bystander CPR by bystander type, and Fig. 3D–F presents the annual trends in the proportion of patients achieving CPC 1–2 at 1-month by bystander type. For family member-witnessed OHCAs, the annual trends in the proportions of both bystander CPR and CPC 1–2 at 1-month significantly increased across all age groups (all *p* for trend <0.001, Fig. 3A and D). However, the proportions of bystander CPR remained at approximately 50% in all age groups. For combined friends/colleagues/passersby-witnessed OHCAs, the annual trends in the proportions of both bystander CPR and CPC 1–2 at 1-month significantly increased among those aged 65–74 and 75–89 years (all *p* for trend <0.001), whereas no significant temporal trend was observed in either bystander CPR or CPC 1–2 among those aged ≥90 years (Fig. 3B and E). However, the number of those aged ≥90 years was relatively small (*n* = 1080, Table 1). For other-witnessed OHCAs, the annual trend in the proportion of bystander CPR significantly increased across all age groups (all *p* for trend <0.001, Fig. 3C). The annual trend in the proportion of CPC 1–2 at 1-month significantly increased only among those aged 65–74 years (*p* for trend <0.05), but not among those aged 75–89 or ≥90 years (Fig. 3F). Among those aged 75–89 and ≥90 years, the proportions of bystander CPR were already high in 2005 and increased further over time, whereas the proportions of CPC 1–2 at 1-month remained low and showed no significant temporal trend. In those aged ≥90 years, the proportion of bystander CPR increased to approximately 80%, but the proportion of CPC 1–2 at 1-month remained at approximately 1%, within the range of 0.471–1.413% (overall, 0.746% [205/27,490], Table 3).

**Fig. 3:**
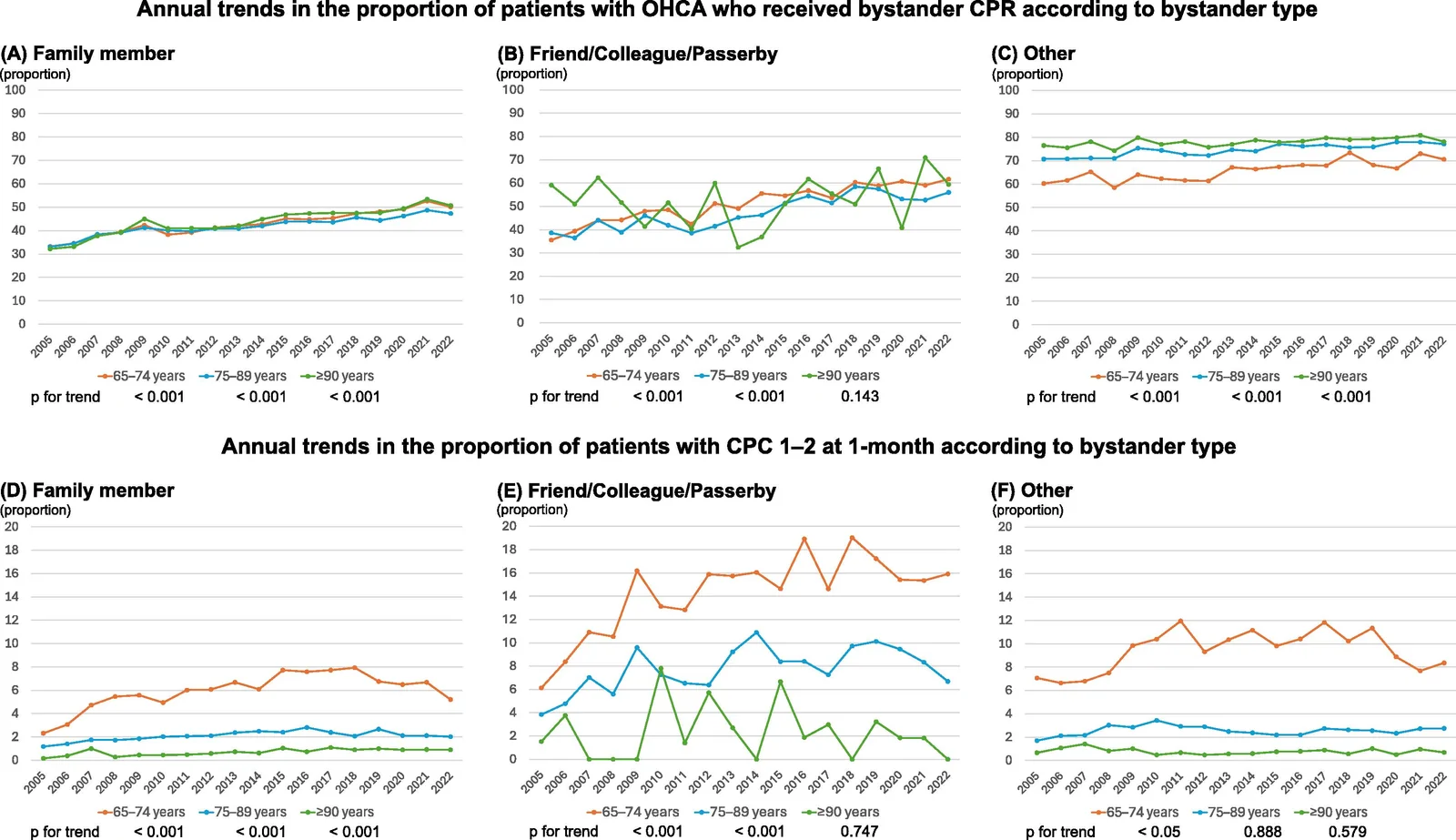
**Annual trends in the proportion of patients with OHCA who received bystander CPR according to bystander type: (A) family member, (B) combined friend/colleague/passerby, and (C) other. Annual trends in the proportion of patients with CPC 1–2 at 1-month according to bystander type: (D) family member, (E) combined friend/colleague/passerby, and (F) other. CPC indicates Cerebral Performance Category scale; CPR, Cardiopulmonary resuscitation; OHCA, Out-of-hospital cardiac arrest**.

**Table 3:** Bystander CPR and CPC 1–2 at 1-month by bystander type and age group.

	**Bystander type (witnessed by)**			***P* value**
	**Family member**	**Combined** **friend/colleague/passerby**	**Other**	
	***n* = 211,398**	***n* = 24,448**	***n* = 87,097**	
65–74 years	*n* = 52,647	*n* = 12,657	*n* = 12,718	
Bystander CPR	22,649 (43.0)	6604 (52.2)	8402 (66.1)	<0.001
CPC 1–2 at 1-month	3133 (6.0)	1853 (14.6)	1211 (9.5)	<0.001

75–89 years	*n* = 124,558	*n* = 10,711	*n* = 46,889	
Bystander CPR	52,596 (42.2)	5163 (48.2)	35,129 (74.9)	<0.001
CPC 1–2 at 1-month	2633 (2.1)	846 (7.9)	1210 (2.6)	<0.001

≥90 years	*n* = 34,193	*n* = 1080	*n* = 27,490	
Bystander CPR	15,282 (44.7)	579 (53.6)	21,518 (78.3)	<0.001
CPC 1–2 at 1-month	250 (0.7)	24 (2.2)	205 (0.7)	<0.001

Values are number of patients (%) unless indicated otherwise.

CPC indicates Cerebral Performance Category scale; CPR, Cardiopulmonary resuscitation.

## Discussion

This nationwide study described temporal trends in bystander CPR and favourable neurological outcomes among older patients with OHCA according to bystander–patient relationship and age. The temporal patterns differed according to these characteristics. In particular, among family member-witnessed OHCAs, the proportions receiving bystander CPR and achieving favourable neurological outcomes increased over time across all age groups. In contrast, among other-witnessed OHCAs aged ≥90 years, bystander CPR increased substantially while favourable neurological outcomes remained low.

In Japan, most older patients with witnessed OHCA are witnessed by family members. It has long been recognized that increasing the proportion of bystander CPR performed by family members is important.^15^, ^16^, ^17^, ^18^ The present study provides a longitudinal descriptive analysis over an 18-year period, focusing on temporal trends stratified by age. Although the proportion receiving bystander CPR among family member-witnessed OHCAs gradually increased over time, it remained at approximately 50% in 2022 across all age groups and thus remains insufficient to date. These findings highlight the importance of continued efforts to promote bystander CPR among family members. The observed temporal increases in bystander CPR and favourable neurological outcomes among family member-witnessed OHCAs suggest that strategies to facilitate bystander CPR by family members warrant further investigation. However, because this study was descriptive and did not account for potential confounding factors, these temporal associations cannot establish a causal relationship between increased bystander CPR and improved favourable neurological outcomes. Emotional stress, panic, or awareness of a patient's prior preferences regarding resuscitation may influence whether family members initiate CPR when witnessing a cardiac arrest.^16^ In addition, family members may be physically unable to perform effective CPR, particularly when the rescuer is an older spouse. The All-Japan Utstein Registry does not contain detailed information on the bystander's age, gender or number of rescuers. These factors may contribute to the relatively low proportion of bystander CPR among family member-witnessed OHCAs. Potential barriers that may prevent family members from performing bystander CPR warrant further investigation.^10^, ^25^, ^26^ CPR training that includes scenarios involving cardiac arrest of a family member at home may be relevant. In addition, increasing awareness of AED locations and how to promptly summon rescuers may be relevant to improving preparedness for bystander CPR.^27^

Other-witnessed OHCAs warrant particular attention. Other-witnessed OHCAs were older, more frequently received bystander CPR, more frequently had emergency calls made before collapse, and had a shorter interval from collapse to bystander CPR (Table 2). These characteristics may be consistent with some patients in this group being residents of nursing homes or healthcare facilities, particularly those aged ≥90 years.^16^, ^28^ However, because the registry does not provide detailed information about the bystander or the location of cardiac arrest, this interpretation remains speculative. Among other-witnessed OHCAs aged ≥90 years, the proportion receiving bystander CPR increased to approximately 80%, whereas the proportion achieving CPC 1–2 remained at approximately 1%. This discrepancy suggests that the temporal increase in bystander CPR was not accompanied by a corresponding increase in favourable neurological outcomes in this group. Several factors, including advanced age, frailty, comorbidities, pre-existing functional status, and characteristics of the cardiac arrest, may contribute to poor neurological outcomes.^28^, ^29^, ^30^ These factors were not available in the registry. Furthermore, although resuscitation guidelines and in-hospital treatment have advanced over time, favourable neurological outcomes among other-witnessed OHCAs aged ≥90 years did not show a temporal increase in our study.^4^, ^31^ The very low proportion of favourable neurological outcomes among other-witnessed OHCAs aged ≥90 years should not be interpreted as indicating that CPR is inappropriate solely because of advanced age. The registry does not contain information on ACP, do not attempt resuscitation orders, patients’ preferences, or family preferences regarding resuscitation. Therefore, we could not determine whether resuscitation was consistent with individual preferences or whether resuscitation was considered appropriate in individual cases. Nevertheless, these findings highlight the importance of ACP and discussions about preferences regarding resuscitation for very old patients, particularly those receiving care in nursing homes or healthcare facilities.^3^, ^28^ Such discussions may help align emergency responses with patients' preferences regarding resuscitation.

Among older patients aged 65–74 and 75–89 years with OHCA witnessed by a combined friend/colleague/passerby, the proportions of both bystander CPR and favourable neurological outcomes increased over time. Although the All-Japan Utstein Registry does not record the location of cardiac arrest, the type of bystander may serve as a proxy for location. A combined friend/colleague/passerby may represent a public place, a family member a private residential setting, and most of others a nursing home or healthcare facility.^28^ Bystander CPR in public settings may be more effective due to earlier recognition, the presence of multiple rescuers to share tasks and maintain compression quality, and greater access to AEDs^32^, ^33^; however, these mechanisms could not be directly evaluated in the present study.

Previous studies using the same registry have examined the association between the bystander–patient relationship and outcomes; however, these studies either excluded other group or dichotomized relationships into family member versus non-family bystander, and none specifically focused on other group.^15^, ^16^, ^17^, ^18^ The present study incorporated more recent data, specifically examined other group, stratified analyses by age, and assessed temporal trends. These analyses provide a descriptive overview of temporal changes in bystander CPR and favourable neurological outcomes across different age and bystander categories in Japan and highlight areas warranting further investigation. The findings may also have relevance to other countries experiencing rapid population aging and may help inform future research and interventions, although prospective or adjusted analyses are needed to determine whether increasing bystander CPR improves neurological outcomes in specific patient subgroups.

### Limitation

This study has several limitations. First, the All-Japan Utstein Registry does not provide detailed information on bystanders categorized as other, and further classification was not possible. Second, detailed information regarding CPR quality, comorbidities, frailty, location of cardiac arrest, and in-hospital management was unavailable. These limitations are inherent to the retrospective study design. Third, although previous studies using the same registry have reported analyses including location data, such data are generally not available and, when they are, are limited to 2013–2015; therefore, they could not be used in the present study.^28^, ^30^, ^26^, ^34^ Fourth, this registry includes data on the bystander–patient relationship, but does not specify the relationship between the patient and the individual who performed BCPR. Fifth, this study did not account for the provision of dispatcher-assisted CPR instructions, which may serve as a proxy for prior CPR training or CPR experience and, consequently, CPR quality.^32^, ^35^ Sixth, the proportions of BCPR and favourable neurological outcome from 2020 onward should be interpreted with consideration of the impact of the COVID-19 pandemic.^36^ Seventh, several resuscitation guideline revisions during the study period, including the 2020 guideline update, may have influenced temporal trends in bystander CPR and favourable neurological outcomes.^4^, ^31^ Eighth, this was a descriptive observational analysis and did not use multivariable modeling to estimate the independent association between bystander CPR and favourable neurological outcomes or to formally assess effect modification by bystander–patient relationship and age. Therefore, the observed temporal concordance between bystander CPR and favourable neurological outcomes should not be interpreted as evidence of a causal effect of bystander CPR.

## Conclusions

In Japan, the proportion of older patients with OHCA receiving bystander CPR increased over time in most bystander–patient relationship and age groups. However, temporal trends in favourable neurological outcomes varied according to bystander–patient relationship and age. These findings suggest that temporal increases in bystander CPR were not uniformly accompanied by improvements in neurological outcomes and warrant further investigation of the factors underlying these differences. The observed temporal trends do not establish a causal relationship between bystander CPR and favourable neurological outcomes.

## Declaration of generative AI and AI-assisted technologies in the manuscript preparation process

During the preparation of this work the authors used ChatGPT in order to assist with language editing and improve readability. After using this tool, the authors reviewed and edited the content as needed and take full responsibility for the content of the published article.

## Funding source

This work was supported by the Japan Society for the Promotion of Science (KAKENHI Grant Number 24K13337).

## CRediT authorship contribution statement

**Akira Funada:** Writing – original draft, Visualization, Validation, Project administration, Methodology, Investigation, Funding acquisition, Formal analysis, Conceptualization. **Yoshikazu Goto:** Writing – review & editing, Validation, Supervision, Conceptualization. **Masayuki Takamura:** Writing – review & editing, Supervision. **Naohiro Yonemoto:** Resources, Data curation. **Tetsuya Matoba:** Writing – review & editing, Conceptualization. **Yoshio Tahara:** Writing – review & editing, Supervision, Conceptualization.

## Declaration of competing interest

The authors declare that they have no known competing financial interests or personal relationships that could have appeared to influence the work reported in this paper.

## References

[1] Matsuyama T., Kitamura T., Kiyohara K. (2018). Assessment of the 11-year nationwide trend of out-of-hospital cardiac arrest cases among elderly patients in Japan (2005–2015). Resuscitation.

[2] Kitamura T., Komukai S., Zha L. (2026). Nationwide trends and future projections of out-of-hospital cardiac arrest in Japan: a Bayesian age-period-cohort model and demographic decomposition analysis. Resuscitation.

[3] Saito Y., Tateishi K., Mori Y. (2025). Out-of-hospital cardiac arrest in septuagenarians, octogenarians, and nonagenarians in Japan: trends and patterns in outcomes. J Am Coll Cardiol.

[4] Japan Resuscitation Council (2021).

[5] Goto Y., Maeda T., Goto Y.N. (2013). Termination-of-resuscitation rule for emergency department physicians treating out-of-hospital cardiac arrest patients: an observational cohort study. Crit Care.

[6] Goto Y., Funada A., Maeda T., Okada H., Goto Y. (2019). Field termination-of-resuscitation rule for refractory out-of-hospital cardiac arrests in Japan. J Cardiol.

[7] Sakamoto A., Inokuchi R., Iwagami M., Hanari K., Tamiya N. (2023). Association between physicians' characteristics and their knowledge, attitudes, and practices regarding advance care planning: a cross-sectional study. BMC Palliat Care.

[8] Inoue M., Hanari K., Hamano J., Gallagher J., Tamiya N. (2019). Current engagement in advance care planning in japan and its associated factors. Gerontol Geriatr Med.

[9] Perman S.M., Elmer J., Maciel C.B. (2024). 2023 American Heart Association focused update on adult advanced cardiovascular life support: an update to the American Heart Association guidelines for cardiopulmonary resuscitation and emergency cardiovascular care. Circulation.

[10] Okada Y., Hong K.J., Lim S.L. (2025). The “invisible ceiling” of bystander CPR in three Asian countries: descriptive study of national OHCA registry. Resuscitation.

[11] Nakahara S., Tomio J., Ichikawa M. (2015). Association of bystander interventions with neurologically intact survival among patients with bystander-witnessed out-of-hospital cardiac arrest in Japan. JAMA.

[12] Wissenberg M., Lippert F.K., Folke F. (2013). Association of national initiatives to improve cardiac arrest management with rates of bystander intervention and patient survival after out-of-hospital cardiac arrest. JAMA.

[13] Yamaguchi T., Nakai M., Kodama T. (2024). Impact of a national initiative to provide civilian cardiopulmonary resuscitation training courses on the rates of bystander intervention by citizens and survival after out-of-hospital cardiac arrest. Resuscitation.

[14] Funada A., Goto Y., Maeda T., Teramoto R., Hayashi K., Yamagishi M. (2016). Improved survival with favorable neurological outcome in elderly individuals with out-of-hospital cardiac arrest in Japan – a nationwide observational cohort study. Circ J.

[15] Akahane M., Tanabe S., Koike S. (2012). Elderly out-of-hospital cardiac arrest has worse outcomes with a family bystander than a non-family bystander. Int J Emerg Med.

[16] Tanaka Y., Maeda T., Kamikura T. (2015). Potential association of bystander**–**patient relationship with bystander response and patient survival in daytime out-of-hospital cardiac arrest. Resuscitation.

[17] Suematsu Y., Zhang B., Kuwano T. (2019). Citizen bystander**–**patient relationship and 1-month outcomes after out-of-hospital cardiac arrest of cardiac origin from the All-Japan Utstein Registry: a prospective, nationwide, population-based, observational study. BMJ Open.

[18] Fujie K., Nakata Y., Yasuda S., Mizutani T., Hashimoto K. (2014). Do dispatcher instructions facilitate bystander-initiated cardiopulmonary resuscitation and improve outcomes in patients with out-of-hospital cardiac arrest? A comparison of family and non-family bystanders. Resuscitation.

[19] Perkins G.D., Jacobs I.G., Nadkarni V.M. (2015). Cardiac arrest and cardiopulmonary resuscitation outcome reports: update of the Utstein Resuscitation Registry Templates for out-of-hospital cardiac arrest: a statement for healthcare professionals from a Task Force of the International Liaison Committee on Resuscitation (American Heart Association, European Resuscitation Council, Australian and New Zealand Council on Resuscitation, Heart and Stroke Foundation of Canada, InterAmerican Heart Foundation, Resuscitation Council of Southern Africa, Resuscitation Council of Asia); and the American Heart Association Emergency Cardiovascular Care Committee and the Council on Cardiopulmonary, Critical Care, Perioperative and Resuscitation. Resuscitation.

[20] Kitamura T., Iwami T., Kawamura T. (2010). Nationwide public-access defibrillation in Japan. N Engl J Med.

[21] Kitamura T., Iwami T., Kawamura T. (2012). Nationwide improvements in survival from out-of-hospital cardiac arrest in Japan. Circulation.

[22] Iwami T., Kitamura T., Kiyohara K., Kawamura T. (2015). Dissemination of chest compression-only cardiopulmonary resuscitation and survival after out-of-hospital cardiac arrest. Circulation.

[23] Nagao K., Nonogi H., Yonemoto N. (2016). Duration of prehospital resuscitation efforts after out-of-hospital cardiac arrest. Circulation.

[24] Ouchi Y., Rakugi H., Arai H. (2017). Redefining the elderly as aged 75 years and older: proposal from the Joint Committee of Japan Gerontological Society and the Japan Geriatrics Society. Geriatr Gerontol Int.

[25] Langlais B.T., Panczyk M., Sutter J. (2017). Barriers to patient positioning for telephone cardiopulmonary resuscitation in out-of-hospital cardiac arrest. Resuscitation.

[26] Matsuyama T., Okubo M., Kiyohara K. (2019). Sex-based disparities in receiving bystander cardiopulmonary resuscitation by location of cardiac arrest in Japan. Mayo Clin Proc.

[27] Hansen S.M., Hansen C.M., Folke F. (2017). Bystander defibrillation for out-of-hospital cardiac arrest in public vs residential locations. JAMA Cardiol.

[28] Okabayashi S., Matsuyama T., Kitamura T. (2019). Outcomes of patients 65 years or older after out-of-hospital cardiac arrest based on location of cardiac arrest in Japan. JAMA Netw Open.

[29] Kitamura T., Morita S., Kiyohara K. (2014). Trends in survival among elderly patients with out-of-hospital cardiac arrest: a prospective, population-based observation from 1999 to 2011 in Osaka. Resuscitation.

[30] Kishimori T., Matsuyama T., Kiyohara K. (2020). Prehospital cardiopulmonary resuscitation duration and neurological outcome after adult out-of-hospital cardiac arrest by location of arrest. Eur Heart J Acute Cardiovasc Care.

[31] Yagi T., Nagao K., Yonemoto N. (2024). Impact of updating the cardiopulmonary resuscitation guidelines on out-of-hospital shockable cardiac arrest: a population-based cohort study in Japan. J Am Heart Assoc.

[32] Takei Y., Nishi T., Matsubara H., Hashimoto M., Inaba H. (2014). Factors associated with quality of bystander CPR: the presence of multiple rescuers and bystander-initiated CPR without instruction. Resuscitation.

[33] Nishi T., Maeda T., Takase K., Kamikura T., Tanaka Y., Inaba H. (2013). Does the number of rescuers affect the survival rate from out-of-hospital cardiac arrests? Two or more rescuers are not always better than one. Resuscitation.

[34] Kiyohara K., Nishiyama C., Matsuyama T. (2019). Out-of-hospital cardiac arrest at home in Japan. Am J Cardiol.

[35] Takahashi H., Tagami T., Suzuki K. (2025). The impact of dispatcher-assisted CPR and prior bystander CPR training on neurologic outcomes in out-of-hospital cardiac arrest: a multicenter study. Resuscitation.

[36] Hosomi S., Zha L., Kiyohara K. (2022). Impact of the COVID-19 pandemic on out-of-hospital cardiac arrest outcomes in older adults in Japan. Resusc Plus.

